# Lipoprotein(a) and Plaque Vulnerability: Mechanistic Insights, Imaging Evidence, and Clinical Implications

**DOI:** 10.3390/biomedicines14102337

**Published:** 2026-10-14

**Authors:** Quanhao Sun, Yajie Wang, Xiaoya Li, Changxin Sun, Xin Bai, Longtao Liu

**Affiliations:** 1 Clinical Medical College, Guang’anmen Hospital, Beijing University of Chinese Medicine, Beijing 100029, China; 13021531569@163.com (Q.S.);; 2 Department of Cardiology, Guang’anmen Hospital, China Academy of Chinese Medical Sciences, Beijing 100053, China; drwyj6887@163.com (Y.W.); lixiaoya0303@foxmail.com (X.L.);; 3 National Clinical Research Center for Chinese Medicine Cardiology, Xiyuan Hospital, China Academy of Chinese Medical Sciences, Beijing 100091, China

**Keywords:** Lipoprotein(a), vulnerable plaque, atherosclerosis, plaque imaging, cardiovascular risk, targeted therapy

## Abstract

Although low-density lipoprotein cholesterol (LDL-C) can now be effectively reduced, substantial residual risk of acute ischemic cardiovascular events persists. Lipoprotein(a) [Lp(a)] is an established causal risk factor for atherosclerotic cardiovascular disease (ASCVD), yet its mechanistic links to plaque vulnerability and their clinical implications remain incompletely understood. Clinical imaging studies have associated elevated Lp(a) with plaque progression and features of vulnerability, including lipid-rich necrotic cores, macrophage accumulation, and thin fibrous caps, suggesting that Lp(a)-associated risk may involve destabilization during plaque progression. This review draws on imaging and pathological evidence to examine the potential mechanisms underlying these associations and their implications for clinical risk stratification and preventive decision-making. The apolipoprotein(a) [apo(a)] component unique to Lp(a), together with the oxidized phospholipids (OxPL) it carries, may contribute to plaque vulnerability by sustaining vascular inflammation, promoting necrotic core expansion, and influencing fibrous cap stability and intraplaque neovascularization. Integrating circulating Lp(a) levels with plaque imaging features and baseline cardiovascular risk may help identify individuals at high risk in primary prevention and evaluate residual risk in secondary prevention, informing earlier or more intensive efforts to manage modifiable risk factors. Alongside established preventive measures, therapies aimed at lowering Lp(a) may provide an additional approach to reducing residual risk. In trials conducted to date, substantial reductions in Lp(a) have been observed with antisense oligonucleotides, small interfering RNAs, and oral small-molecule inhibitors; some agents have demonstrated sustained effects and favorable safety profiles. However, whether therapies targeting Lp(a) reduce cardiovascular event risk remains to be established through robust evidence from large cardiovascular outcome trials.

## 1. Introduction

Over the past three decades, atherosclerotic diseases—including ischemic heart disease, peripheral artery disease, and ischemic stroke—have shown a sustained rise in global age-standardized incidence [1]. The pathological progression of atherosclerosis (AS) is a dynamic and multistep process that leads, through plaque rupture or erosion, to most acute cardiovascular events [2]. Muller et al. [3] first proposed the concept of a “vulnerable plaque” in 1989, equating it with that of a “rupture-prone plaque.” Such plaques typically have a large lipid-rich necrotic core beneath a thin fibrous cap with sparse smooth muscle cells (SMCs) and abundant macrophage infiltration, accompanied by abnormal intraplaque neovascularization. With advances in the understanding of unstable plaque pathophysiology and the development of imaging technologies, in vivo assessment of culprit lesions in acute coronary syndrome (ACS) has revealed that eroded plaques—pathologically defined by a small lipid core, a thick fibrous cap, and a mild inflammatory response—account for more than one-third of ACS cases. The rise in non-ST-segment elevation myocardial infarction incidence has been linked particularly to eroded plaques [4,5]. However, the evidence concerning whether lipoprotein(a) [Lp(a)] is associated with plaque erosion remains limited. The few available studies using optical coherence tomography (OCT) have not found a significant association of circulating Lp(a) with plaque erosion [6], whereas evidence linking higher Lp(a) levels to lipid-rich plaques and thin-cap fibroatheroma (TCFA) has been more consistent [7]. Accordingly, the following discussion focuses primarily on how Lp(a) relates to rupture-prone plaques.

Approximately 20% of people worldwide have elevated Lp(a) levels (>125 nmol/L or >50 mg/dL) [8]. In Lp(a), apolipoprotein(a) [apo(a)] is attached by a disulfide bond to apolipoprotein B-100 (apoB-100) on a low-density lipoprotein (LDL)-like particle [9]. Measurements using quantitative lipoprotein electrophoresis show that cholesterol carried within Lp(a) particles makes a substantial contribution to laboratory-reported low-density lipoprotein cholesterol (LDL-C) values [10]. Unlike LDL, however, Lp(a) has additional proinflammatory, proatherogenic, and prothrombotic activity mediated by apo(a), oxidized phospholipids (OxPL), and other components [11,12,13]. Current evidence indicates that Lp(a)-associated cardiovascular risk is relatively independent of, and additive to, the risk associated with LDL-C. A meta-analysis of six statin trials involving 27,658 participants found that elevated Lp(a) remained associated with atherosclerotic cardiovascular disease (ASCVD) risk even among participants who achieved low LDL-C levels; those with elevations in both Lp(a) and LDL-C had the highest risk [14]. At the plaque level, Shishikura et al. [15] found that patients with coronary artery disease (CAD) who had both LDL-C < 70 mg/dL and Lp(a) < 50 mg/dL showed the lowest near-infrared spectroscopy–derived maximum 4 mm lipid-core burden index across the four lipid groups; a similar pattern was observed in a further analysis using LDL-C < 55 mg/dL. Plasma Lp(a) levels show high heritability, with estimates spanning approximately 51% to 98% across populations and much of the variation between individuals associated with genetic differences at the LPA gene locus [16,17,18,19]. Thus, assessing the actual plaque-related risk linked to elevated Lp(a) may be more relevant to current clinical practice than simply identifying elevated circulating levels.

However, Lp(a) testing remains substantially underutilized worldwide, and well-established strategies for managing Lp(a)-related cardiovascular risk are still lacking [20,21]. Fang et al. [22] have reviewed the association of Lp(a) with high-risk coronary plaques, and Greco et al. [23] have examined Lp(a) as an ASCVD risk factor and therapeutic target. Drawing on mechanistic, pathological, and multimodal imaging evidence, we examine Lp(a) in relation to plaque vulnerability, focusing on the association of elevated Lp(a) with rupture-prone phenotypes and considering whether these phenotypes could refine the clinical interpretation and stratification of Lp(a)-related cardiovascular risk. We also address unresolved inconsistencies in the available evidence and the limitations of its clinical translation.

## 2. Search Strategy

We searched PubMed, Embase, and Web of Science Core Collection for records published between January 1974 and September 2026, using Medical Subject Headings (MeSH) in PubMed, Emtree terms in Embase, and corresponding free-text terms. Search terms included Lipoprotein(a), Apoprotein(a), Atherosclerosis, Plaque, Atherosclerotic, Inflammation, Neovascularization, Pathologic, Risk Assessment, Primary Prevention, and Secondary Prevention, together with terms related to apo(a)/Lp(a) structure and composition, plaque progression and vulnerability, OxPL, multimodality plaque imaging, and Lp(a)-lowering therapies. Relevant preclinical (in vitro and animal), translational and genetic, human pathological and imaging, and clinical studies (randomized, non-randomized, prospective, and retrospective) were considered eligible. Relevant guidelines, consensus statements, and review articles were also considered, and reference lists were checked to identify additional studies. Studies were selected for discussion according to their relevance to Lp(a)-related plaque vulnerability or its clinical implications and the clarity of their design and outcomes. When multiple studies addressed the same question, we gave priority to original reports with directly relevant outcomes while retaining studies with divergent findings or complementary populations, vascular beds, imaging modalities, or experimental models. For the emerging therapeutics section, ClinicalTrials.gov was additionally searched to identify ongoing registered trials and trials for which peer-reviewed results were not yet available.

## 3. Elevated Lipoprotein(a): Clinical Imaging Evidence Across Plaque Progression and Vulnerability

Plaque burden and plaque vulnerability are interrelated features of atherosclerosis that may coexist and evolve during plaque progression, disruption, and healing. Kinoshita et al. [24] assessed 990 plaques in 419 patients using coronary computed tomography angiography (CCTA) combined with OCT. Among patients with ACS, greater plaque volume was linked to more frequent macrophage accumulation and a larger lipid arc, whereas among those with chronic coronary syndrome (CCS), it was linked to a higher layered plaque index [24]. Over 12 months of follow-up in patients with ST-segment elevation myocardial infarction, ruptured plaques at the culprit lesion site showed significant healing, with the median lumen area decreasing from 7.5 mm^2^ to 3.6 mm^2^ [25], suggesting that plaque healing may contribute to increased plaque burden. Autopsy analyses have likewise linked greater luminal stenosis to a higher number of healed plaque ruptures [26]. Together, these observations suggest that increasing plaque burden and changes in plaque vulnerability are interconnected and that their relationship may vary by clinical presentation [24,26].

Longitudinal findings indicate that Lp(a) remains relatively stable within individuals: 25 years after measurement at ages 9–24 years, 94% of those with Lp(a) ≥ 30 mg/dL still exceeded this threshold, and elevated levels in childhood and young adulthood were associated with a higher risk of ASCVD events in adulthood [27,28]. Together, these findings suggest that sustained Lp(a) elevation may promote plaque formation and progression over time. Its oxidized phospholipid cargo and proinflammatory effects may also be associated with vulnerable plaque phenotypes, with imaging features such as lipid-rich necrotic cores, fibrous caps that are thin or ruptured, and intraplaque hemorrhage [29,30,31,32]. Long-term imaging studies provide supporting evidence. In a 10-year follow-up of 804 participants without cardiovascular disease at baseline, Duan et al. [33] found that Lp(a) ≥ 50 mg/dL was associated with overall carotid plaque progression and, in those initially free of plaque, the development of ultrasound-defined vulnerable plaque. In a separate study, 267 patients with suspected CAD had a second CCTA scan after a median interval of 10.2 years [34]. The study defined elevated Lp(a) as ≥125 nmol/L and found that higher concentrations were significantly associated with greater plaque burden progression, the presence of low-attenuation noncalcified plaque, and higher perivascular adipose tissue attenuation, which may reflect pericoronary inflammation [34]. Taken together, these results support associations of elevated Lp(a) with plaque burden and vulnerability, with a possible contribution from inflammatory pathways. Using OCT in 202 patients with ACS, Di Muro et al. [35] observed more pronounced macrophage infiltration and a higher prevalence of lipid-rich plaques among those with Lp(a) ≥ 300 mg/L than among those with lower levels. Human and ex vivo investigations have associated elevated Lp(a) with greater inflammation of the arterial wall and increased recruitment of peripheral blood mononuclear cells to that site. Monocytes from individuals with elevated Lp(a) levels also showed greater transendothelial migration and more pronounced proinflammatory cytokine responses [11]. These observations further support an association between elevated Lp(a) and inflammatory activity, complementing the imaging evidence linking Lp(a) to vulnerable plaque features. Potential mechanisms that may explain these associations are discussed in subsequent sections.

However, reported associations of Lp(a) with plaque burden and composition have varied across studies. Kaiser et al. [2] studied 191 patients with advanced stable CAD, of whom 160 underwent a second CCTA scan 12 months later. Low-attenuation plaque volume increased more in patients with elevated Lp(a), whereas no statistically significant between-group differences were found in the changes in total, calcified, or noncalcified plaque volumes [2]. In the PROSPECT II substudy of 865 patients with recent myocardial infarction (MI), higher Lp(a) levels were independently associated with focal high-risk lesions combining high local plaque burden with high lipid core burden, while no independent association was found with total coronary plaque volume or the global coronary lipid core burden index [29]. These associations may also vary across patient subgroups. In a sex-stratified analysis of the PARISK cohort with symptomatic carotid stenosis, Lp(a) was associated with intraplaque hemorrhage (IPH) in women and with stenosis severity in men. These observations require validation in larger studies [32]. Overall, the evidence suggests associations of Lp(a) with both plaque burden and plaque vulnerability. However, these should not be regarded as interchangeable outcomes. Comparisons across studies should account for differences in study populations, vascular territories, imaging modalities, and follow-up duration (Table 1). Current evidence does not support delineating distinct stages in which Lp(a) is associated with only plaque burden or only plaque vulnerability.

## 4. Structural and Compositional Features of Lp(a): Links to Plaque Vulnerability

Lp(a) assembly begins in hepatocytes, where apo(a) interacts transiently with apoB-100 in a lysine-dependent manner; an extracellular covalent disulfide bond then forms between Cys-4057 of apo(a) and Cys-4326 of apoB-100 to yield a stable circulating Lp(a) particle [9]. Circulating Lp(a) concentrations are closely related to apo(a) isoform size, whereas the protein and lipid composition of the particle may influence its accumulation in the vascular wall and its local inflammatory effects [36,37,38]. Considering these two features helps clarify Lp(a)’s relationship to plaque vulnerability.

**Figure 1:**
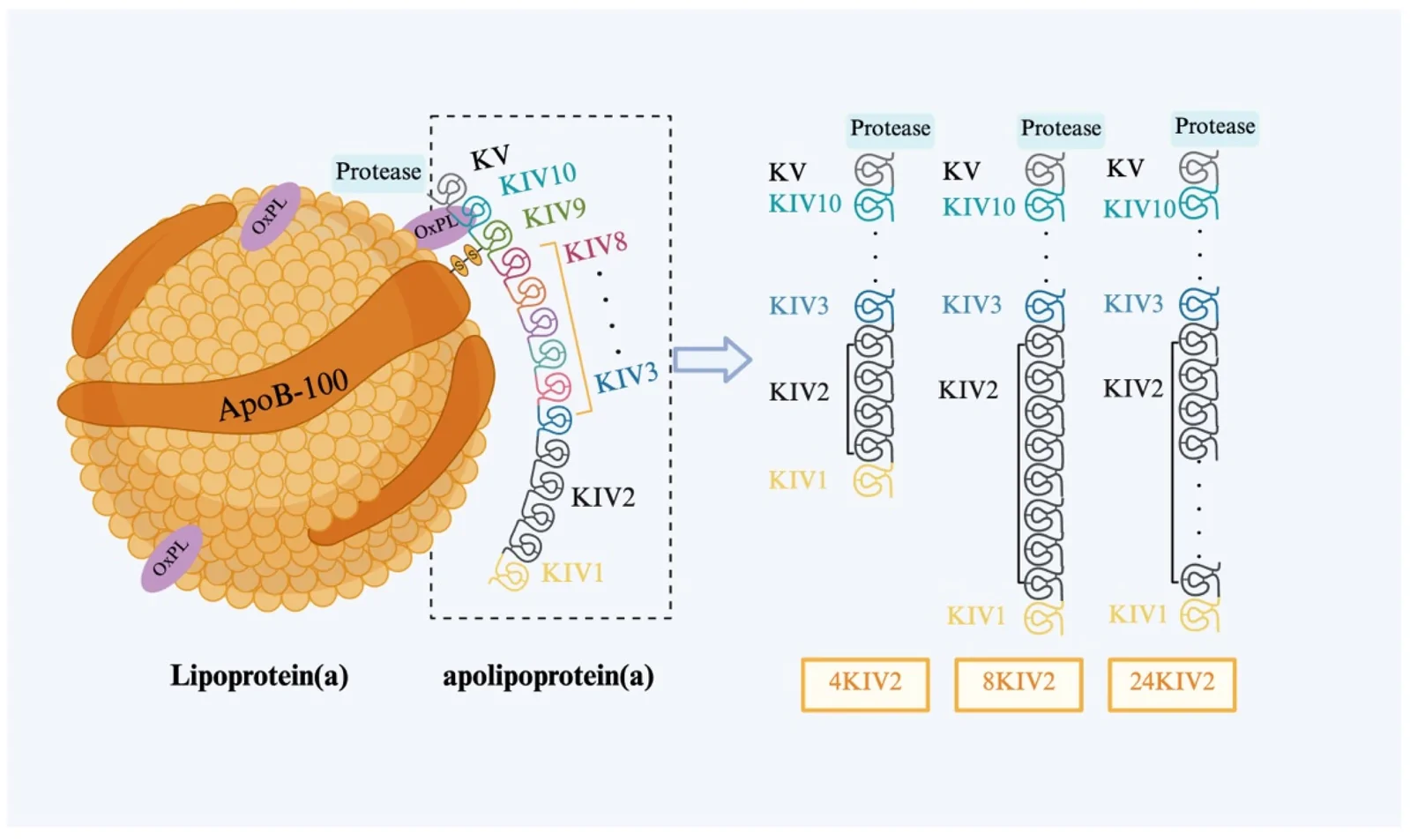
Structure and Heterogeneity of Lp(a) Particles. The variable number of KIV2 repeats accounts for the marked size heterogeneity of apo(a); 4KIV2, 8KIV2, and 24KIV2 denote apo(a) isoforms containing 4, 8, and 24 KIV2 repeats, respectively. Abbreviations: Lp(a), lipoprotein(a); OxPL, oxidized phospholipids; ApoB-100, apolipoprotein B-100; apo(a), apolipoprotein(a); KV, kringle V; KIV, kringle IV. Created in BioRender. Sun, Q. (2026) https://biorender.com/6yi72cm (accessed on 5 October 2026).

Variation in apo(a) size is one aspect of Lp(a)’s structural heterogeneity. Apo(a) has 10 distinct kringle IV (KIV) domain types, numbered KIV-1 to KIV-10. Between individuals, KIV-2 repeat number varies substantially and determines apo(a) isoform size; it is generally inversely related to plasma Lp(a) concentrations [36,39,40] (Figure 1). Kinetic analyses in humans have found that Lp(a) production rates are higher with smaller apo(a) isoforms [36], helping to explain the higher circulating concentrations associated with these isoforms. Kamstrup et al. [40] found that fewer KIV-2 repeats were associated with higher plasma Lp(a) concentrations and greater MI risk. Clarke et al. [41] subsequently linked two LPA gene risk alleles, intronic rs10455872 and coding rs3798220, to fewer KIV-2 repeats, smaller apo(a) isoforms, and higher Lp(a) concentrations. Several studies have investigated whether plaque characteristics vary with apo(a) isoform size. In a study of 80 patients undergoing carotid endarterectomy, women with unstable plaques had smaller predominant apo(a) isoforms in serum, higher serum Lp(a) concentrations, and greater plaque Lp(a) content than women with stable plaques. The corresponding differences in these Lp(a)-related measures were not statistically significant among men [42]. In the PARISK study, apo(a) KIV-2 repeat count was significantly inversely related to plasma Lp(a) concentrations, but no significant association emerged with carotid plaque characteristics on MRI or multidetector computed tomography angiography [32]. Taken together, studies consistently show that apo(a) isoform size is closely related to circulating Lp(a) concentrations; whether isoform size affects plaque vulnerability after accounting for Lp(a) concentrations remains unclear.

Beyond differences in isoform size, apo(a) lysine binding is also implicated in the accumulation of Lp(a) within the vascular wall and in effects associated with OxPL. In animal experiments, Lp(a) with a defective lysine-binding site showed less accumulation in the vascular wall than Lp(a) with an intact site, despite comparable circulating levels [37]. Among plasma lipoproteins, Lp(a) is the predominant carrier of OxPL bearing a phosphocholine headgroup [43,44]. Cell-based experiments found that specific mutations of the strong lysine-binding site in apo(a) KIV-10 markedly diminished E06-detectable OxPL on apo(a) and reduced its ability to induce IL-8 expression in macrophages [38]. These findings suggest that this structural feature is linked to OxPL-related inflammatory effects. OxPL can act as damage-associated molecular patterns, engaging surface receptors on immune and non-immune cells. For instance, CD36 recognizes certain OxPL species and can cooperate with innate immune receptors such as Toll-like receptors (TLRs) to initiate intracellular inflammatory signaling [11,45]. Immunostaining shows widespread apo(a) and OxPL in plaques at all stages, with especially marked accumulation in the thin fibrous caps and underlying necrotic cores of vulnerable lesions, such as TCFA and ruptured plaques [46]. The biological effects associated with OxPL also involve their metabolites. Lipoprotein-associated phospholipase A2 (Lp-PLA2), or plasma platelet-activating factor acetylhydrolase, is associated with several lipoprotein classes. A fraction is bound to Lp(a) and participates in the metabolism of OxPL carried by these particles [47,48,49]. When Lp-PLA2 hydrolyzes certain OxPL species, the resulting lysophosphatidylcholine (LPC) and oxidized free fatty acids exert pro-inflammatory and pro-apoptotic effects. In addition, Lp-PLA2 expression is high in the necrotic cores of vulnerable and ruptured plaques and in surrounding macrophages, and the enzyme is also detected in apoptotic macrophages. These findings suggest a possible contribution of Lp-PLA2 to necrotic core–associated inflammation and apoptosis and, consequently, a close link to plaque destabilization and rupture [50,51].

Other lipid components of Lp(a) also have potential biological activity. Lipidomic analyses have shown that, relative to LDL obtained from the same donors, Lp(a) has a lower relative content of polyunsaturated fatty acids, particularly n-3 polyunsaturated fatty acids, and a higher n-6/n-3 fatty acid ratio. The authors proposed that these differences in fatty acid composition could contribute to the stronger inflammatory response elicited by Lp(a) [52]. Dzobo et al. [53] found that Lp(a) contained more diacylglycerols (DGs) than LDL and also detected LPC, a precursor of lysophosphatidic acid, in Lp(a). Cell-based experiments showed that certain DG species promoted inflammatory responses in monocytes and enhanced their transendothelial migration; lysophosphatidic acid also enhanced monocyte transendothelial migration [53]. These findings suggest that these lipids may contribute to plaque pathology through their effects on monocyte recruitment and inflammatory responses, although their link to plaque vulnerability remains largely supported by such indirect evidence.

Lp(a) also contains apoB-100, a protein found in LDL and some triglyceride-rich lipoproteins. An apoB-based genetic analysis of UK Biobank data estimated a roughly 6.6-fold stronger association with CAD risk per Lp(a) particle than per LDL particle [54]. Among patients with stable CAD and elevated Lp(a), Ezhov et al. [55] found that higher total apoB-100 levels correlated with greater necrotic core volume on virtual histology intravascular ultrasound. However, the study did not distinguish the lipoprotein sources of apoB-100. In another study of men undergoing elective PCI, serum IgG against the native apoB-100 p45 peptide correlated inversely with the necrotic plaque component and positively with the fibrous component among those with less than one month of prior statin treatment [56]. These findings raise the possibility that apoB-100 and related immune responses are associated with plaque composition but are insufficient to establish an independent role for apoB-100 within Lp(a) particles in plaque vulnerability.

Taken together, circulating Lp(a) levels influence vascular wall exposure to Lp(a) particles and their lipid cargo; the lysine-binding function of apo(a) is associated with Lp(a) accumulation in the vascular wall; and particle-associated bioactive lipids can contribute to inflammatory responses. These structural and compositional features help explain the relationship between Lp(a) and plaque vulnerability at the molecular level.

## 5. Mechanisms Linking Lp(a) to Plaque Vulnerability

### 5.1. Inflammatory Cell Recruitment and Activation

Monocytes, as key drivers of vascular inflammation, play a crucial role in nearly all stages of AS. Monocytes comprise a heterogeneous population whose distinct subsets are defined by CD14 and CD16 expression [57,58]. In Krychtiuk et al.’s study of stable CAD, patients with Lp(a) > 50 mg/dL had a greater proportion of intermediate monocytes (CD14^++^CD16^+^) than those with Lp(a) ≤ 50 mg/dL. OxPL/apoB levels were also positively correlated with the proportion of intermediate monocytes [59], suggesting that Lp(a) may be associated with differences in monocyte subset distribution. Intermediate monocytes show increased expression of genes involved in oxygen radical production, decreased expression of antioxidant-related genes, and higher basal reactive oxygen species (ROS) levels, consistent with an inflammatory and activated phenotype [60]. An optical coherence tomography study linked higher circulating intermediate monocyte levels to thinner fibrous caps and a greater frequency of TCFA [61]. OxPL may also contribute to the monocyte activation and vascular wall inflammation associated with Lp(a). Studies in humans and in cell culture indicate that, in individuals with high Lp(a), circulating monocytes show persistent activation, greater endothelial adhesion, and increased transendothelial migration [11,62]. This phenotype is characterized by marked upregulation of markers including C-C chemokine receptor 7, CD11b, CD11c, and CD29. Neutralization of OxPL carried by Lp(a) significantly attenuates this sustained pro-inflammatory activation. Lp(a) can also promote monocyte migration towards the vascular endothelium by modulating endothelial cell metabolism. Specifically, OxPL upregulates 6-phosphofructo-2-kinase/fructose-2,6-bisphosphatase-3, a key enzyme regulating glycolysis, thereby increasing endothelial glycolytic flux to help meet the energy demands of the inflammatory response [12]. Notably, Lp(a) also appears to enhance myelopoiesis, thereby increasing the supply of pro-inflammatory cells. In vitro, exposure of murine bone marrow cells to Lp(a) increased granulocyte–monocyte progenitor numbers and the production of pro-inflammatory Ly6C^high^ monocytes and macrophages. With continuous Lp(a) exposure for 7 days, bone marrow cells showed greater proliferation and colony formation, along with increased generation of inflammatory myeloid cells. Blockade of Lp(a)-associated OxPL with E06 abolished the myelopoietic effect of Lp(a), suggesting that OxPL are key mediators of this response [63].

Recently, Rosenson et al. [62] identified a mechanism by which Lp(a) induces monocyte tissue factor (TF) expression via the TLR2/nuclear factor kappa B (NF-κB) signaling axis, thereby linking inflammation and thrombosis. TF not only serves as a key regulator initiating the coagulation protease cascade but also amplifies inflammatory responses [64]. Consistent with findings from Sachetto et al. [64], Sotiriou et al. [65] demonstrated that Lp(a) functions as a ligand for the leukocyte integrin αMβ2 (Mac-1), activating NF-κB, inducing TF expression in monocytes, and promoting prothrombotic factor production. Clinical studies have demonstrated that patients with high Lp(a) exhibit concomitant elevations in plasma TF and pro-inflammatory cytokines, including CCL28 and IL-17D [62], suggesting a vicious cycle of thrombosis and inflammation that may contribute to a prothrombotic milieu. Lipid components carried by Lp(a), including DGs and lysophosphatidic acid, also have pro-inflammatory potential. Studies of patients with elevated Lp(a) have found markedly elevated plasma concentrations of DGs and lysophosphatidic acid, both of which are specifically enriched in Lp(a) particles. Cell culture studies further show that DGs stimulate monocytes to release the pro-inflammatory cytokines IL-6, IL-8, and IL-1β and promote their transendothelial migration, with NLR family pyrin domain containing 3 (NLRP3) inflammasome activation contributing in part to these effects [53]. Taken together, these observations suggest that Lp(a) may facilitate monocyte entry into the arterial wall and sustain a local inflammatory microenvironment through several linked mechanisms involving inflammatory signaling and cell recruitment, thereby contributing to vulnerable plaque development and progression. Beyond TF induction, in vitro studies have shown that Lp(a) and recombinant apo(a) inhibit fibrin-dependent activation of plasminogen (PLG) by tissue-type plasminogen activator and enhance platelet aggregation elicited by a low concentration of the thrombin receptor–activating peptide SFLLRN [66,67]. These effects may compound the consequences of plaque vulnerability by favoring thrombosis after plaque rupture.

Macrophages are highly heterogeneous and plastic cells. Lp(a) can modulate macrophage phenotype and function, thereby participating in both the initiation and maintenance of inflammatory responses. A significant colocalization of Lp(a) with M1-type macrophages has been observed within AS plaques [53]. Carotid plaque tissues from patients with high Lp(a) are specifically enriched with resident macrophages and inflammatory macrophages. Resident macrophages highly express TLR4 and triggering receptor expressed on myeloid cells, contributing to pathogen recognition and inflammation initiation, whereas inflammatory macrophages highly express P-selectin and genes associated with the NLRP3 inflammasome pathway, driving robust inflammatory responses [53]. The dynamic evolution of macrophages is central to AS plaque progression and vulnerability, and their spatial aggregation patterns may contain important pathological information. A recent retrospective OCT study of 404 patients showed that larger macrophage clusters within coronary plaques (angular extent > 72°) were significantly associated with features of plaque vulnerability [68]. Research indicates that as plaques progress, macrophages at the lesion site are replaced by newly recruited macrophages derived from circulating monocytes [69]. Despite their distinct origins, these macrophages converge in their transcriptional profiles after entering the plaque microenvironment and becoming foam cells, exhibiting shared gene expression programs driven in part by local microenvironmental cues [69]. Macrophage foam cell formation has traditionally been attributed largely to the uptake of oxidized LDL through scavenger receptors [70]. However, macrophages within human AS plaques express the very low-density lipoprotein receptor (VLDL-R), which can directly mediate Lp(a) uptake, leading to foam cell formation independently of classical scavenger receptors [71,72]. In cultured cells, VLDL-R expression was not suppressed by sterol-mediated feedback, while VLDL-R overexpression promoted lipid accumulation and foam cell formation after exposure to β-VLDL [71,73,74]. Together, these findings raise the possibility that VLDL-R-mediated Lp(a) uptake could continue despite cellular lipid accumulation and contribute to foam cell formation during sustained exposure to elevated Lp(a).

**Figure 2:**
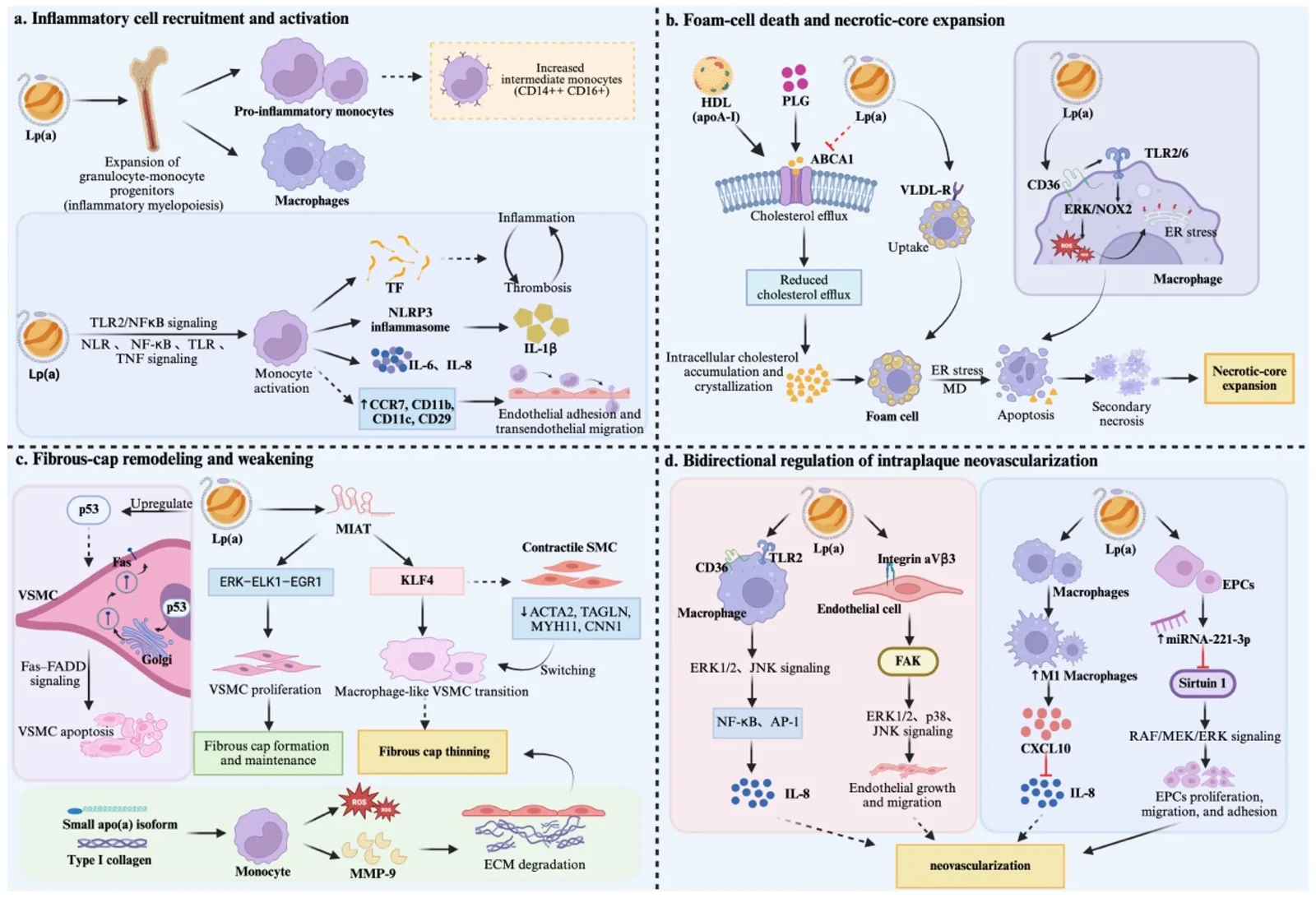
Mechanisms Linking Lipoprotein(a) to Plaque Vulnerability. (**a**) Lp(a) promotes inflammatory myelopoiesis, monocyte activation, inflammatory-cell recruitment, and pro-inflammatory and prothrombotic signaling. (**b**) Lp(a) may impair cholesterol efflux and enhance lipoprotein uptake, promoting foam-cell formation, macrophage apoptosis, secondary necrosis, and necrotic-core expansion. (**c**) Lp(a) may influence fibrous-cap stability through VSMC proliferation, apoptosis and phenotypic modulation, together with MMP-9–mediated ECM degradation. (**d**) Lp(a) may bidirectionally regulate intraplaque neovascularization through pro-angiogenic effects on macrophages and endothelial cells and anti-angiogenic effects involving M1 macrophages and EPC dysfunction. Solid arrows indicate experimentally supported mechanisms, whereas dashed arrows indicate proposed or indirect mechanistic relationships for which direct Lp(a)-specific evidence remains limited; blunt-ended lines indicate inhibition. Abbreviations: ABCA1, ATP-binding cassette transporter A1; ACTA2, actin alpha 2, smooth muscle; AP-1, activator protein 1; apo(a), apolipoprotein(a); apoA-I, apolipoprotein A-I; CCR7, C-C chemokine receptor type 7; CD, cluster of differentiation; CNN1, calponin 1; CXCL10, C-X-C motif chemokine ligand 10; ECM, extracellular matrix; EGR1, early growth response 1; ELK1, ETS transcription factor ELK1; EPCs, endothelial progenitor cells; ER, endoplasmic reticulum; ERK, extracellular signal-regulated kinase; FADD, Fas-associated death domain protein; FAK, focal adhesion kinase; Fas, Fas cell surface death receptor; HDL, high-density lipoprotein; IL, interleukin; JNK, c-Jun N-terminal kinase; KLF4, Krüppel-like factor 4; Lp(a), lipoprotein(a); MD, mitochondrial dysfunction; MEK, mitogen-activated protein kinase/ERK kinase; MIAT, myocardial infarction associated transcript; MMP-9, matrix metalloproteinase-9; MYH11, myosin heavy chain 11; NF-κB, nuclear factor kappa B; NLR, NOD-like receptor; NLRP3, NLR family pyrin domain containing 3; NOX2, NADPH oxidase 2; p38, p38 mitogen-activated protein kinase; p53, tumor protein p53; PLG, plasminogen; RAF, rapidly accelerated fibrosarcoma kinase; ROS, reactive oxygen species; SMC, smooth muscle cell; TAGLN, transgelin; TF, tissue factor; TLR, Toll-like receptor; TNF, tumor necrosis factor; VLDL-R, very-low-density lipoprotein receptor; VSMC, vascular smooth muscle cell. Created in BioRender. Sun, Q. (2026) https://biorender.com/kgazhmq (accessed on 5 October 2026).

In addition to macrophages arising from monocytes, SMCs can also undergo phenotypic switching towards a macrophage-like state. Fasolo et al. [75] confirmed that Lp(a) can upregulate myocardial infarction-associated transcript (MIAT) expression, which promotes the transdifferentiation of SMCs into inflammatory macrophage-like cells, a process dependent on Krüppel-like factor 4 (KLF4) activation. Interestingly, MIAT can also promote the proliferation of SMCs through ERK/ELK1/EGR1 signaling, resulting in fibrous cap thickening and enhanced plaque stability [75]. Thus, Lp(a)-induced MIAT may contribute to the progression of plaques while also influencing their stability through distinct effects on SMCs (Figure 2).

### 5.2. Necrotic Core Expansion

The necrotic core is an amorphous, soft atheromatous region composed of cellular debris, cholesterol crystals (CCs), and inflammatory mediators. Its lack of collagen matrix support renders plaques highly prone to rupture. Among statin-treated patients with angina, up to 62% still exhibit progression of the necrotic core in non-culprit lesions, with serum Lp(a) level serving as a strong independent predictor of such progression [76]. Using OCT, Xue et al. [77] found that CCs were present in the culprit lesions of 39% of ACS patients, with the only difference in lipid profiles between patients with and without CCs being Lp(a) levels. However, the mechanism by which Lp(a) contributes to CC formation has not yet been experimentally demonstrated. Beyond physical factors influencing cholesterol crystallization kinetics and volume, we hypothesize that Lp(a) may disrupt cholesterol homeostasis and promote the deposition of free CCs.

One key mechanism of necrotic core enlargement is diminished cholesterol efflux capacity (CEC). Under cholesterol-loading conditions, one major pathway for macrophage cholesterol efflux involves ATP-binding cassette transporter A1 (ABCA1), which facilitates the transfer of cholesterol to lipid-poor apolipoprotein A-I (apoA-I), the principal apolipoprotein of high-density lipoprotein (HDL); this transfer represents an early step in reverse cholesterol transport [78]. Although plasminogen (PLG) is not an apolipoprotein, Pamir et al. [79] found that it can also facilitate cholesterol efflux through ABCA1. Subsequently, Tavori et al. [80] reported that total serum CEC was 24.1% to 30.6% lower in patients with Lp(a) levels > 50 mg/dL than in those with low Lp(a) levels. Structural homology with PLG offers a possible explanation for this reduction: Lp(a) may compete with PLG for binding to ABCA1 and thus significantly lower CEC [79,80]. Impaired CEC can promote intracellular cholesterol accumulation and foam cell formation. Excess free cholesterol can trigger endoplasmic reticulum stress and mitochondrial dysfunction, thereby favoring macrophage apoptosis [81,82,83]. In vitro evidence suggests that Lp(a)-associated OxPL can induce apoptosis in macrophages undergoing endoplasmic reticulum stress through a CD36–TLR2/6–ROS-dependent pathway. The involvement of CD36/TLR signaling in macrophage apoptosis and plaque necrosis is further supported by animal studies [84]. Inadequate clearance of apoptotic cells can lead to secondary necrosis, which allows lipid-rich cellular contents to accumulate and exacerbates local inflammation, thereby further promoting necrotic core formation [85]. An enlarged necrotic core directly reduces the plaque’s mechanical stability and may also increase protease release, including the release of matrix metalloproteinases (MMPs), which degrade collagen in the fibrous cap and lead to cap thinning and a higher risk of plaque rupture [76,86] (Figure 2).

### 5.3. Fibrous Cap Remodeling and Weakening

The marked plasticity of vascular SMCs during AS development permits a shift from a quiescent, contractile state to a synthetic phenotype capable of proliferating, migrating, and synthesizing matrix [87]. This phenotypic switch may serve a compensatory role. On the one hand, SMCs may contribute to positive vascular remodeling by migrating and remodeling the extracellular matrix (ECM), allowing the vessel wall to expand outwards and thereby limiting luminal narrowing during certain stages of plaque growth [88,89]. On the other hand, some SMCs that undergo phenotypic switching can migrate into the fibrous cap region, re-express differentiation-related genes, and deposit ECM components, including collagen, thereby forming a dense fibrous cap that helps maintain plaque stability [90]. Komai et al. [91] demonstrated in vitro that Lp(a) and its oxidized form stimulate vascular SMC proliferation via ERK/MAPK pathway activation. However, this in vitro study could not reproduce the specific stages of AS progression.

Current evidence suggests that Lp(a) may indirectly modulate vascular SMC apoptosis, phenotypic switching towards a macrophage-like state, and intracellular lipid accumulation or foam cell formation [75,92,93], potentially increasing plaque instability. Iwabayashi et al. [94] found that Lp(a) induced ROS generation and premature endothelial cell senescence and increased the expression of p53 and p21, a cyclin-dependent kinase inhibitor downstream of p53. These findings suggest that Lp(a) may promote premature cellular senescence through DNA damage related to oxidative stress, together with cell-cycle arrest. Notably, p53 is also an important regulator of vascular SMC apoptosis. p53 has been shown to promote Fas (CD95) trafficking from the Golgi complex to the cell surface and activate apoptotic signaling dependent on Fas and Fas-associated death domain protein (FADD), inducing SMC apoptosis [95]. Plaque-derived vascular SMCs are particularly susceptible to p53-mediated apoptosis, and this heightened susceptibility may be related to their proliferative state [96]. As previously mentioned, Lp(a) upregulates MIAT expression, indirectly activating KLF4 transcription [75]. Consistent with findings by Shankman et al. [97], vascular SMC-specific knockout of KLF4 markedly suppressed their transdifferentiation into macrophage-like and mesenchymal stem cells, resulting in reduced plaque volume, thickened fibrous caps, increased α-smooth muscle actin-positive cells, and decreased apoptosis. Further investigations revealed that KLF4 directly binds to G/C repressor elements in the promoter regions of SMC marker genes (e.g., Acta2, Tagln), inhibiting their transcription while simultaneously activating genes involved in inflammation, phagocytosis, and migration [97]. In addition, impaired autophagy in SMCs can promote their apoptosis and is an important contributor to fibrous cap thinning [98,99]. However, whether this process is linked to Lp(a) remains unconfirmed and warrants further investigation.

Simultaneously, synthetic SMCs secrete ECM, while MMPs mediate its degradation, thereby reducing fibrous cap thickness and making plaque rupture more likely. Sabbah et al. [100] demonstrated that small apo(a) isoforms, in synergy with type I collagen, significantly stimulate monocytes to secrete ROS and MMP-9, an effect that diminishes as the number of KIV-2 repeats increases. Elevated MMP-9 expression has been confirmed to promote ECM degradation, enhance inflammatory cell infiltration, and increase intraplaque neovascularization (IPN), collectively contributing to plaque destabilization [101,102].

Notably, the effects of Lp(a) on MMP-9 may vary according to the pathological context. Clinical studies have documented substantially elevated circulating MMP-9 concentrations among individuals with ACS. On the day of symptom onset, plasma MMP-9 levels in the unstable angina and acute MI groups were approximately three and two times the levels in healthy controls, respectively [103]. Huang et al. [104] found that, under acute inflammatory conditions, Lp(a) could competitively inhibit PLG activation and reduce the formation of active MMP-9, thereby limiting acute ECM degradation. However, neither pro-MMP-9 nor total MMP-9 levels changed significantly, suggesting that this effect primarily involves MMP-9 activation rather than expression. By contrast, among individuals with stable CAD and Lp(a) concentrations of at least 50 mg/dL, MMP-9 levels varied inversely with the proportions of fibrous and fibrofatty tissue and were significantly related to plaque vulnerability [55]. These observations raise the possibility that Lp(a) upregulates MMP-9 biosynthesis in the setting of chronic inflammation, although the causal relationship remains to be clarified. Conversely, improvements in systemic metabolic and inflammatory status, such as those observed during glucagon-like peptide-1 receptor agonist therapy, have been accompanied by parallel declines in circulating concentrations of Lp(a) and MMP-9 [105].

### 5.4. Intraplaque Neovascularization

A retrospective analysis involving individuals with carotid stenosis identified an independent positive association between Lp(a) concentration and IPN presence. After multivariable adjustment, the odds ratio for IPN was 1.305 per 10 mg/dL increment in Lp(a) (95% CI, 1.045–1.628; *p* = 0.019) [106]. IPN is a pathological process driven by intraplaque hypoxia and chronic inflammation; neovessels arising from the lumen and extending into the plaque interior are particularly destructive [107,108]. These neovessels are structurally abnormal, with loose endothelial junctions, making them highly prone to leakage or rupture, inducing recurrent IPH. Following vessel rupture, free cholesterol from erythrocyte membranes promotes necrotic core expansion and the formation of pro-inflammatory CCs, while oxidative stress mediated by hemoglobin and iron ions further induces macrophage recruitment and activation, stimulating MMP secretion. Consequently, the fibrous cap thins and the plaque’s mechanical strength decreases [109,110,111,112]. At the molecular mechanism level, OxPL carried by apo(a) is recognized by macrophage surface receptors (e.g., CD36/TLR2). This activates the ERK1/2 and c-Jun N-terminal kinase (JNK) signaling pathways, synergistically upregulating the activity of transcription factors NF-κB and activator protein 1 (AP-1), and driving IL-8 transcription [38]. In human coronary atherosclerotic plaques, IL-8 expression is significantly increased at both the protein and mRNA levels; both signals colocalize with the endothelial cell marker factor VIII–related antigen and with macrophages [113]. Animal experiments further confirmed that implanting human coronary plaque tissue into the rat cornea induced a strong angiogenic response, which was completely inhibited by IL-8 neutralizing antibodies [113], suggesting IL-8 may mediate a potential link between Lp(a)-related inflammatory signaling and IPN. Furthermore, Liu et al. [114] found that the interaction of apo(a) with integrin αVβ3 activates focal adhesion kinase (FAK) and downstream MAPK signaling involving ERK1/2, p38, and JNK, while reducing transforming growth factor-β (TGF-β) expression and stimulating endothelial cell proliferation and migration. Further in vitro evidence indicates that FAK/ERK1/2 signaling is involved in promoting endothelial cell migration and tube-like structure formation [115], suggesting that this pathway may contribute to neovascularization. Although the direct involvement of TGF-β in IPN remains unexplored, Mallat et al. [116] found that inhibition of TGF-β signaling increased plaque inflammation and reduced collagen content, favoring a vulnerable plaque phenotype.

Previous studies have shown that proteolytic cleavage of PLG generates angiostatin, which exhibits antiangiogenic activity [117]. Given the high structural homology of apo(a) with PLG, some studies have suggested a possible antiangiogenic effect of Lp(a). Currently, evidence supporting this view mainly stems from observations that Lp(a) impairs endothelial cell function as well as the function of endothelial progenitor cells (EPCs). For instance, Zhang and colleagues [118] reported that Lp(a) upregulates miR-221-3p expression in EPCs; miR-221-3p targets and inhibits Sirtuin 1, impeding RAF/MEK/ERK pathway activation and consequently suppressing EPC proliferation, migration, adhesion, and angiogenic capacity. Furthermore, Lp(a) can promote macrophage polarization toward the M1 phenotype, while CXCL10 secreted by M1 macrophages can exert antiangiogenic effects by inhibiting IL-8 activity [119,120].

Taken together, Lp(a) may exert complex, bidirectional effects on IPN, promoting it through inflammatory and oxidative signaling while exerting inhibitory effects by impairing endothelial repair and EPC function (Figure 2).

## 6. Lp(a) and Cardiovascular Disease Prevention

### 6.1. Lp(a) Testing and Risk Stratification: From Plaque Evidence to Preventive Decisions

Lp(a) elevation is established as an independent ASCVD risk factor; genetic evidence supports its causal role [121]. As mechanistic and imaging evidence linking Lp(a) to plaque vulnerability accumulates, interest has grown in combining Lp(a) measurement with plaque imaging to refine risk assessment, although the incremental value of this approach for clinical decision-making remains to be established. Within current guideline-based practice, overall risk assessment should account for Lp(a); the magnitude of Lp(a) elevation together with the individual’s baseline risk should inform how intensively LDL-C is lowered and how other modifiable risk factors are managed. The ESC/EAS reiterated in its 2025 focused dyslipidemia update the recommendation to consider at least one Lp(a) measurement over the course of every adult’s life [122]. The 2026 ACC/AHA multisociety dyslipidemia guideline assigned Class I recommendations (Level of Evidence B-NR) both to this testing approach and to cascade Lp(a) testing among first-degree relatives of people with elevated Lp(a), premature ASCVD, or familial hypercholesterolemia [123].

Measuring Lp(a) can help evaluate cardiovascular event risk among patients with ASCVD and should also aid early identification of risk before clinical events occur. In their large study with extended follow-up, Berman and colleagues [124] observed that the likelihood of major adverse cardiovascular events (MACE) rose as Lp(a) levels increased in the secondary prevention cohort, then plateaued at approximately 112 nmol/L. In the primary prevention group, risk continued to increase as Lp(a) rose, and the highest risk was observed at levels ≥ 216 nmol/L. In its 2025 focused update, the ESC/EAS recommended using Lp(a) to modify risk assessment, particularly when reclassifying individuals whose risk is moderate or close to a threshold for treatment decisions [122]. The 2026 ACC/AHA guideline also emphasized that an elevated Lp(a) concentration calls for more intensive efforts to lower LDL-C, together with efforts to control other modifiable risk factors [123]. Thus, within this guideline-based framework, a prudent clinical approach is to consider the additional risk associated with Lp(a) beyond that captured by traditional risk factors, taking the primary or secondary prevention setting into account when determining whether modifiable cardiovascular risk factors warrant earlier or more intensive management, rather than basing treatment decisions solely on a given Lp(a) concentration. Combining Lp(a) measurement with plaque imaging is a promising approach to further characterizing risk, but its incremental value for selecting or intensifying preventive treatment beyond guideline-based assessment remains to be established.

### 6.2. Primary Prevention

In individuals without diagnosed ASCVD, assessment of Lp(a)-associated risk should consider baseline risk, long-term prognosis, and subclinical atherosclerosis. A study following 1215 individuals without cardiovascular disease at baseline for 25 years found that, after stratification using the CUORE risk charts, the predictive value of Lp(a) for long-term cardiovascular mortality was mainly evident in high-risk individuals and women at intermediate risk [125]. The findings suggest that the prognostic relevance of Lp(a) in primary prevention may vary depending on baseline risk and sex. A large study covering participants from primary as well as secondary prevention cohorts found that each further 50 nmol/L rise in Lp(a) corresponded to significantly greater risks of both MACE and MI, independent of the baseline concentration of high-sensitivity C-reactive protein (hs-CRP) [126]. Among primary prevention participants in EPIC-Norfolk, Lp(a), LDL-C, and hs-CRP were each independent predictors of MACE, and event risk increased additively when all three were elevated [127]. Thus, in primary prevention, systemic inflammation reflected by hs-CRP may contribute to higher absolute risk alongside elevated Lp(a), but low hs-CRP levels do not eliminate the long-term risk associated with Lp(a). Because circulating hs-CRP does not fully capture vascular wall inflammation, this association does not preclude local proinflammatory effects of Lp(a)/OxPL. Future studies should incorporate repeated hs-CRP measurements and upstream inflammatory markers such as IL-6 in ethnically diverse cohorts with sufficient numbers of events to clarify the interaction between inflammation and Lp(a) and determine whether baseline inflammatory status predicts benefit from Lp(a)-lowering therapy. Beyond circulating biomarkers, imaging-based assessment of subclinical atherosclerosis may further refine risk stratification for those whose Lp(a) concentration is elevated. For participants with coronary artery calcium (CAC) scores ≥ 100 and Lp(a) concentrations ≥ 50 mg/dL, Mehta and colleagues [128] reported a 10-year cumulative ASCVD incidence of 23.2%; among those with CAC scores < 100, the association between elevated Lp(a) and greater risk was not statistically significant. These findings suggest that Lp(a) and CAC provide complementary prognostic information in primary prevention. However, a low 10-year risk should not be taken to rule out longer-term risk associated with Lp(a). Whether the combined assessment of Lp(a) and CAC improves preventive treatment decisions beyond guideline-based risk assessment remains to be established.

The possibility that Lp(a) may inhibit fibrinolysis and interact with platelet function has heightened interest in whether aspirin use could improve outcomes in the primary prevention setting for people whose Lp(a) concentrations are elevated. Genetic subgroup analyses and evidence from observational studies suggest that aspirin could reduce cardiovascular event risk among individuals whose Lp(a) is elevated [129,130]. However, a net clinical benefit remains unproven, and current evidence is inadequate to recommend aspirin routinely for primary prevention in people with elevated Lp(a). Decisions about whether to use aspirin should therefore consider each person’s baseline risk of ASCVD, age, and bleeding risk. In a recent expert opinion, the Polish Lipid Association (PoLA) suggested that, after a thorough evaluation of bleeding risk, aspirin at a low dose could be considered as a primary prevention option for individuals at high or very high cardiovascular risk whose Lp(a) concentration exceeds 50 mg/dL [131].

### 6.3. Secondary Prevention

Among patients with established ASCVD, the clinical focus shifts from identifying the risk of a first event to assessing residual risk despite standard therapy. In light of the imaging evidence discussed above, Lp(a)-associated residual risk may reflect not only plaque burden but also vulnerable plaque phenotypes. However, whether tailoring Lp(a)-related treatment to these imaging phenotypes improves clinical outcomes remains unproven. In secondary prevention populations, higher Lp(a) levels show a graded association with recurrent MACE risk. Madsen et al. [132] estimated that achieving 20% and 40% reductions in MACE risk over five years would require respective Lp(a) decreases of 50 mg/dL and 99 mg/dL, suggesting that clinically meaningful risk reduction may require a substantial decrease in Lp(a). A prospective study of 12,064 patients undergoing percutaneous coronary intervention found that a baseline Lp(a) level above 30 mg/dL was independently associated with recurrent ischemic events; the association persisted among patients receiving statins [133]. Current therapies primarily targeting LDL-C generally have a limited impact on Lp(a) concentrations. However, without definitive cardiovascular outcome evidence for Lp(a)-specific therapies, intensive reduction of LDL-C and comprehensive efforts to manage additional modifiable risk factors, including blood pressure, glycemia, and smoking, remain the mainstays of secondary prevention. For individuals with elevated Lp(a), the 2026 ACC/AHA multisociety guideline recommends that modifiable risk factors be optimized early. For patients with clinical ASCVD, a monoclonal antibody directed against proprotein convertase subtilisin/kexin type 9 (PCSK9) with demonstrated cardiovascular benefit is recommended when either non–high-density lipoprotein cholesterol or LDL-C remains above target despite maximally tolerated statin therapy. A retrospective study of patients with CAD who had elevated Lp(a) and an inadequate response to pharmacotherapy found that regular long-term lipoprotein apheresis significantly reduced Lp(a) concentrations and was linked to a 71.43% reduction in the revascularization rate [134]. However, the clinical use of lipoprotein apheresis remains limited by its strict eligibility criteria, invasiveness, and high cost, as well as the predominantly observational nature of the available evidence.

### 6.4. Emerging Pharmacotherapies Targeting Lp(a)

Kinetic studies indicate that plasma Lp(a) concentrations depend mainly on apo(a) production rates, with clearance contributing relatively little, although the low-density lipoprotein receptor may participate in the removal of Lp(a) under certain conditions [36,135,136]. Accordingly, two strategies are central to current Lp(a)-specific drug development: targeting LPA mRNA with antisense oligonucleotides (ASOs) or small interfering RNAs (siRNAs) to suppress hepatic apo(a) synthesis and directly interfering with the binding of apo(a) to apoB-100, thereby disrupting the assembly of Lp(a) particles.

Pelacarsen is an ASO targeting apo(a). Its 5′ end is covalently conjugated to N-acetylgalactosamine (GalNAc), which is efficiently recognized by asialoglycoprotein receptors on the hepatocyte surface, enabling selective uptake into hepatocytes. After entering the nucleus, pelacarsen base-pairs with apo(a) mRNA and recruits ribonuclease H1 to degrade the target mRNA, thereby inhibiting apo(a) synthesis [137,138]. A phase II study enrolled 286 individuals with established cardiovascular disease and baseline Lp(a) ≥ 60 mg/dL; the magnitude of Lp(a) lowering increased with pelacarsen dose, and the largest mean decline was 80%. Injection-site reactions were the principal safety signal, while liver and kidney function and platelet counts were generally comparable to those in the placebo group [139]. The phase III Lp(a)HORIZON study (NCT04023552) enrolled 8323 individuals with established cardiovascular disease and baseline Lp(a) ≥ 70 mg/dL; they were assigned at random to pelacarsen (80 mg subcutaneously each month) or matching placebo, with MACE as the primary endpoint [140]. In a topline company announcement dated 4 September 2026, Novartis reported that Lp(a)HORIZON did not meet its primary cardiovascular endpoint [141]. Detailed peer-reviewed trial results were not available at the time of writing.

Lepodisiran consists of an siRNA linked to GalNAc for uptake by hepatocytes, where it targets LPA mRNA and reduces apo(a) synthesis, thereby limiting the supply of apo(a) for Lp(a) assembly. In the phase I dose-ascending study, Lp(a) fell more at higher doses; following a single subcutaneous 608 mg dose, the median level remained 94% below baseline on day 337, and lepodisiran was generally well tolerated in this small cohort. Plasma lepodisiran became undetectable within 48 h, yet Lp(a) lowering persisted; its Dicer-substrate siRNA structure and GalNAc-mediated delivery to hepatocytes are established features, but their contribution to this prolonged effect remains uncertain [142]. The phase II ALPACA study randomized 320 adults with baseline Lp(a) of at least 175 nmol/L. Pooling the two regimens that began with 400 mg yielded a placebo-adjusted, time-averaged change in Lp(a) of −93.9 percentage points over days 60–180. None of the serious adverse events was attributed by the investigators to the assigned treatment. Injection-site reactions were generally mild and became more frequent with increasing dose, affecting 12% of those given 400 mg at both scheduled visits [143]. The phase III ACCLAIM-Lp(a) study (NCT06292013) is testing whether subcutaneous lepodisiran reduces MACE in adults with Lp(a) ≥ 175 nmol/L who have either established ASCVD or a high risk of their first ASCVD event; cardiovascular outcome results have not yet been reported [144].

Olpasiran is a standard siRNA modified with 2′-fluoro/methoxy groups, sharing a similar mechanism of action with lepodisiran. A phase I dose-escalation trial included 64 participants with elevated Lp(a). A single subcutaneous dose of olpasiran lowered Lp(a) levels by 71–97%; at doses ≥ 9 mg, the reductions persisted for several months, and olpasiran was generally well tolerated [145]. Among 281 patients with established ASCVD and baseline Lp(a) > 150 nmol/L, the phase II OCEAN(a)-DOSE trial found that higher olpasiran doses produced greater reductions in Lp(a). At 36 weeks, the placebo-adjusted mean changes in Lp(a) were −97.4% for the 75 mg group and −101.1% for the 225 mg group, with both regimens administered at 12-week intervals. Adverse-event frequencies were similar across trial groups, while drug-related adverse reactions were predominantly mild injection-site reactions [146]. Moreover, olpasiran markedly reduced OxPL bound to apoB, and the magnitude of this reduction was strongly correlated with the decline in Lp(a). However, the study found no significant effect of olpasiran on systemic inflammatory markers, including hs-CRP and high-sensitivity IL-6 [147]. In OCEAN(a)-Outcomes (NCT05581303), an ongoing phase III trial, investigators are assessing whether subcutaneous olpasiran administered every 12 weeks reduces major cardiovascular events among patients with prior ASCVD and Lp(a) ≥ 200 nmol/L [148].

Zerlasiran, another siRNA conjugated to GalNAc, targets the mRNA encoding apo(a) in hepatocytes. In the phase I study, a single subcutaneous injection of zerlasiran yielded maximum median Lp(a) reductions of 96% with 300 mg and 98% with 600 mg. Following multiple doses, Lp(a) concentrations remained 60–90% below baseline on day 201, suggesting prolonged Lp(a) lowering [149]. In ALPACAR-360 (phase II), 178 participants with stable ASCVD and baseline Lp(a) ≥ 125 nmol/L were enrolled; those assigned to zerlasiran received it subcutaneously every 24 weeks at 300 mg or 450 mg, or every 16 weeks at 300 mg. Across the first 36 weeks, the placebo-adjusted time-averaged decrease in Lp(a) exceeded 80% for each regimen; the largest median reduction reached 97.2%, and the effects persisted through week 60. No serious adverse event reported during the study was considered related to zerlasiran [150].

Beyond injectable nucleic acid therapies, preventing Lp(a) particle formation with an oral agent offers an alternative therapeutic strategy. Muvalaplin is an orally administered small molecule that prevents Lp(a) particle formation by interfering with the initial noncovalent association of apo(a) with apoB-100. Among healthy participants with Lp(a) ≥ 30 mg/dL in the phase I randomized, placebo-controlled trial, 14 days of once-daily oral muvalaplin at doses of at least 100 mg lowered Lp(a) by 63–65% compared with placebo. Mild reductions in PLG activity were observed in the two highest-dose groups, peaking at approximately 14% with the 500 mg dose before returning to baseline after treatment discontinuation. PLG concentrations and other fibrinolytic parameters showed no clear dose- or time-dependent changes [151]. A subsequent phase II trial further evaluated its Lp(a)-lowering efficacy and safety in 233 participants whose Lp(a) was ≥175 nmol/L and who had ASCVD, diabetes, or familial hypercholesterolemia. After 12 weeks, once-daily muvalaplin led to placebo-adjusted reductions in intact Lp(a) particle concentrations of 47.6%, 81.7%, and 85.8% at doses of 10, 60, and 240 mg, respectively, without affecting PLG activity [152]. Currently, the phase III MOVE-Lp(a) trial (NCT07157774) is evaluating cardiovascular outcomes; its results are pending [153]. Key clinical trials of these emerging Lp(a)-lowering therapies are summarized in Table 2.

Overall, published phase I and II trials have reported Lp(a) reductions ranging from approximately 80% to over 95% with selected regimens. However, differences in dosing, follow-up duration, and outcome measures preclude direct comparisons across studies. More importantly, Lp(a) lowering is a pharmacodynamic endpoint and does not, by itself, establish cardiovascular benefit; whether these therapies reduce cardiovascular events requires confirmation in adequately powered randomized cardiovascular outcome trials.

## 7. Conclusions and Future Perspectives

Lp(a) is an established causal risk factor for ASCVD, but its specific relationship with plaque vulnerability remains incompletely understood. Plaque burden and vulnerability are interrelated manifestations of the dynamic course of AS and may coexist and change during plaque progression, disruption, and healing. Human imaging and pathological studies suggest that elevated Lp(a) is associated with plaque progression and features of vulnerability linked to rupture, but these associations do not establish a direct causal role in increasing plaque vulnerability. Mechanistic studies suggest that Lp(a), apo(a), and their associated OxPL may contribute to plaque vulnerability by influencing inflammatory cell recruitment, lipid accumulation, necrotic core formation, fibrous cap remodeling, and intraplaque neovascularization. However, the strength of evidence varies across mechanisms, and support for the mechanistic links between Lp(a) and plaque vulnerability in humans still comes largely from experimental studies and indirect human evidence. These findings are insufficient to establish Lp(a) as a direct driver of plaque vulnerability.

In clinical practice, current guidelines increasingly emphasize the additional information provided by Lp(a) measurement for cardiovascular risk assessment. In those with elevated Lp(a), modifiable cardiovascular risk factors should be addressed early, and consideration should be given to more intensive LDL-C lowering in light of overall cardiovascular risk. Combining Lp(a) concentrations with plaque imaging features may help identify individuals at high risk. However, it remains to be established whether this combined assessment improves risk prediction and whether treatment adjustments based on it reduce cardiovascular events.

Existing therapies primarily aimed at lowering LDL-C typically have little effect on Lp(a) concentrations, prompting efforts to develop agents directed specifically at Lp(a). Clinical trials have shown substantial Lp(a) lowering with ASOs, siRNAs, and small-molecule inhibitors. However, the observed lowering of Lp(a) does not, by itself, establish a reduction in cardiovascular events. Whether these targeted therapies provide clinical benefit, which populations may benefit, and their long-term safety will need to be assessed in light of the full results of cardiovascular outcomes trials. Whether they improve plaque stability and how such changes relate to clinical outcomes requires further investigation.

## Figures and Tables

**Table 1:** Clinical imaging studies of lipoprotein(a) in relation to plaque burden and vulnerability.

Study	Vascular Territory	Design and Imaging Interval	Population	Imaging Modality	Lp(a) Units and Exposure	Plaque Endpoints	Main Findings	Main Limitations
Kaiser et al. 2022 [2]	Coronary	Longitudinal Prospective observational analysis; 12 months	Stable multivessel CAD; *n* = 191 at baseline; 160 with repeat CCTA	Serial quantitative CCTA	mg/dL ≥70 vs. <70; additional continuous analysis	Change in total, calcified, noncalcified and low-attenuation plaque volumes	Low-attenuation plaque increased by 26.2 vs. −0.7 mm^3^ (*p* = 0.020). Changes in other plaque volumes did not differ significantly between groups.	Single center; selected advanced CAD population; short imaging interval; stented vessels excluded; residual confounding.
Nurmohamed et al. 2024 [34]	Coronary	Longitudinal Prospective serial imaging; median 10.2 years	Suspected CAD without known CAD at baseline; *n* = 267	AI-assisted quantitative CCTA	nmol/L ≥125; continuous effects per doubling; measured at follow-up only	Change in PAV and plaque components; presence of low-density plaque; PCAT attenuation	Per doubling of Lp(a): adjusted PAV progression +0.32 percentage points per 10 years (95% CI 0.04–0.60); follow-up low-density plaque OR 1.21 (1.01–1.45). Higher PCAT attenuation was also associated with Lp(a).	Selection and survivor bias; Lp(a) measured only at follow-up; scanner changes; stented or poor-quality vessels excluded. PCAT attenuation is an indirect inflammation marker.
Di Muro et al. 2025 [35]	Coronary Culprit vessel	Cross-sectional Retrospective analysis of prospectively collected data; one pre-PCI examination	ACS undergoing OCT-guided PCI; *n* = 202	OCT	mg/L ≥300 vs. <300	Lipid-rich plaque, macrophages, TCFA, plaque rupture and erosion	High Lp(a) was associated with lipid-rich plaque (adjusted OR 4.41, 95% CI 2.35–8.28) and more macrophages. TCFA was numerically more frequent but not statistically significant (*p* = 0.061); rupture and erosion did not differ significantly.	Single-center selected ACS cohort; culprit-vessel assessment; small sample; pre-OCT lesion manipulation may affect morphology; no progression assessment.
Erlinge et al. 2025 [29] PROSPECT II	Coronary Untreated nonculprit lesions	Cross-sectional imaging analysis within a prospective cohort; one baseline examination	Recent MI; *n* = 865	Three-vessel NIRS–IVUS	nmol/L Continuous effects per 100 nmol/L	Pancoronary plaque volume and LCBI; focal PB ≥ 70% and maxLCBI_4_mm ≥ 324.7	Per 100 nmol/L, adjusted OR for a lesion with both high PB and high maxLCBI_4_mm was 1.65 (95% CI 1.11–2.46). No independent association with global plaque volume or lipid burden.	No serial imaging; acute-MI population; culprit and flow-limiting treated lesions excluded; only 21 participants had Lp(a) > 125 nmol/L.
Duan et al. 2023 [33] CMCS Beijing	Carotid Bilateral CCA, bifurcation and ICA	Longitudinal Prospective cohort; 10 years	CVD-free community cohort; *n* = 804; 649 without baseline plaque	Serial high-resolution B-mode ultrasound	mg/dL ≥50 vs. <50; additional continuous and categorical analyses	Plaque progression; incident plaque and ultrasound-defined vulnerable plaque; plaque area and vulnerability score	Lp(a) ≥ 50 mg/dL: adjusted RR 1.41 (95% CI 1.21–1.64) for plaque progression. Among those without baseline plaque, RR 1.93 (1.54–2.41) for incident vulnerable plaque.	Ultrasound has limited sensitivity for vulnerability; Chinese community cohort; few participants with Lp(a) ≥ 50 mg/dL (*n* = 35); attrition and residual confounding.
van Dam-Nolen et al. 2021 [32] PARISK	Carotid Ipsilateral artery	Cross-sectional Baseline analysis within a prospective cohort; one imaging assessment	Recent TIA or minor stroke; ipsilateral 30–69% stenosis; *n* = 182	MDCTA (*n* = 161) and 3-T multicontrast MRI (*n* = 171)	mg/dL Continuous effects per 10 mg/dL	Stenosis, calcification and ulceration; LRNC, IPH, thin or ruptured cap, maximum wall area	Per 10 mg/dL, adjusted ORs were 1.07 (95% CI 1.00–1.15) for LRNC and 1.07 (1.01–1.14) for thin/ruptured cap. Sex-stratified associations included IPH in women and greater stenosis in men.	Cross-sectional associations; selected symptomatic mild-to-moderate stenosis; small sex subgroups; incomplete imaging across modalities; no temporal or causal inference.

Notes: Lp(a) units are retained as reported. Because the relationship between these units varies with apo(a) isoform size and assay characteristics, no universal conversion factor is applicable. Abbreviations: ACS, acute coronary syndrome; AI, artificial intelligence; CAD, coronary artery disease; CCA, common carotid artery; CCTA, coronary computed tomography angiography; CMCS, Chinese Multi-Provincial Cohort Study; CVD, cardiovascular disease; ICA, internal carotid artery; IPH, intraplaque hemorrhage; LCBI, lipid core burden index; Lp(a), lipoprotein(a); LRNC, lipid-rich necrotic core; maxLCBI_4_mm, maximum LCBI over a 4 mm segment; MDCTA, multidetector computed tomography angiography; MI, myocardial infarction; MRI, magnetic resonance imaging; NIRS–IVUS, near-infrared spectroscopy–intravascular ultrasound; OCT, optical coherence tomography; OR, odds ratio; PAV, percent atheroma volume; PB, plaque burden; PCAT, pericoronary adipose tissue; PCI, percutaneous coronary intervention; RR, relative risk; TCFA, thin-cap fibroatheroma; TIA, transient ischemic attack.

**Table 2:** Clinical trials of emerging lipoprotein(a)-lowering therapies.

Study and Phase	Drug	Population	Route and Dosing Schedule	Lp(a) Reduction and Assessment Time	Safety Findings
Tsimikas et al. 2020 [139] Phase 2 NCT03070782	Pelacarsen	*n* = 286; established CVD; Lp(a) ≥ 60 mg/dL	SC: 20, 40 or 60 mg q4wk; 20 mg q2wk; or 20 mg weekly; treatment 6–12 months	At 6 months, mean within-group reductions: 35%, 56%, 72%, 58% and 80%, respectively; placebo 6%.	Injection-site reactions: 27% vs. 6% with placebo. SAEs: 10% vs. 2%, without a dose trend. No significant between-group differences in platelet counts, liver or renal measures.
Cho et al. 2025 [140] Lp(a)HORIZON Phase 3 NCT04023552	Pelacarsen	*n* = 8323; established CVD; Lp(a) ≥ 70 mg/dL; prespecified ≥ 90 mg/dL population	SC 80 mg monthly vs. placebo	Design paper reports no observed phase 3 reduction. Subsequent topline announcement reports lowering without a numerical estimate [142].	No phase 3 safety results in the design paper; detailed safety data were not provided in the cited topline announcement.
Nissen et al. 2023 [142] Phase 1 NCT04914546	Lepodisiran	*n* = 48; no CVD; Lp(a) ≥ 75 nmol/L or ≥30 mg/dL	SC single dose: 4, 12, 32, 96, 304 or 608 mg	Maximum median reductions: 41%, 59%, 76%, 90%, 96% and 97%, respectively. At 608 mg, median reduction remained 94% on day 337.	Mild injection-site reactions; transient aminotransferase and creatine kinase elevations. One unrelated serious traumatic injury.
Nissen et al. 2025 [143] ALPACA Phase 2 NCT05565742	Lepodisiran	*n* = 320; age ≥ 40 years; Lp(a) ≥ 175 nmol/L; mixed CV risk, not exclusively established ASCVD	SC 16, 96 or 400 mg at baseline and day 180; additional arm: 400 mg at baseline, placebo on day 180	Primary placebo-adjusted time-averaged reductions, days 60–180: 40.8, 75.2 and 93.9 percentage points, respectively (400 mg arms pooled). Two 400 mg doses: 94.8 percentage points over days 30–360.	Injection-site reactions: 0–12% across active groups. Liver enzymes > 3× ULN in 7/251 treated participants, without bilirubin elevation; resolved. No investigator-attributed treatment-related SAEs.
Koren et al. 2022 [145] Phase 1 NCT03626662	Olpasiran	*n* = 64; cohorts with Lp(a) 70–199 or ≥200 nmol/L; some higher-Lp(a) participants had CVD	SC single dose: 3, 9, 30, 75 or 225 mg in 70–199 nmol/L cohorts; 9 or 75 mg in ≥200 nmol/L cohorts	Maximum mean reductions: 71–97% in 70–199 nmol/L cohorts and 76–91% in ≥200 nmol/L cohorts; nadir on days 43–71.	No SAEs in olpasiran recipients; one injection-site reaction; no clinically relevant laboratory abnormalities.
O’Donoghue et al. 2022 [146] OCEAN(a)-DOSE Phase 2 NCT04270760	Olpasiran	*n* = 281; ASCVD; Lp(a) > 150 nmol/L	SC 10, 75 or 225 mg q12wk, or 225 mg q24wk; 48-week treatment period, final dose at week 36	Week 36 placebo-adjusted mean changes: −70.5%, −97.4%, −101.1% and −100.5%, respectively. Values beyond −100% reflect the adjusted treatment contrast.	Overall AE incidence similar across groups; mainly mild localized injection-site reactions, with some treatment discontinuations; no Hy’s law cases.
Nissen et al. 2024 [149] Phase 1 NCT04606602	Zerlasiran	Lp(a) ≥ 150 nmol/L; 14 in extended single-dose follow-up; 36 with stable ASCVD in multiple-dose cohorts	SC single 300 or 600 mg; or two doses of 200 mg q4wk, 300 mg q8wk or 450 mg q8wk	Single-dose maximum median reductions: 96%/98%. Multiple-dose maxima: 97%/98%/99%; reductions on day 201: 60%/90%/89%, respectively.	Common mild-to-moderate injection-site reactions; transient CRP increases. No SAEs or drug-induced liver injury in multiple-dose cohorts.
Nissen et al. 2024 [150] ALPACAR-360 Phase 2 NCT05537571	Zerlasiran	180 randomized; 178 treated; stable ASCVD; Lp(a) ≥ 125 nmol/L	SC 450 mg q24wk ×2; 300 mg q16wk ×3; or 300 mg q24wk ×2	Primary placebo-adjusted time-averaged reductions through week 36: 85.6%, 82.8% and 81.3%, respectively. These are distinct from the maximum median reduction.	Mostly mild, transient injection-site reactions; two isolated liver enzyme elevations > 3× ULN without bilirubin elevation. No investigator-attributed treatment-related SAEs.
Nicholls et al. 2023 [151] Phase 1 NCT04472676	Muvalaplin	*n* = 114 healthy adults: 55 single-dose participants; 59 multiple-dose participants with Lp(a) ≥ 30 mg/dL	Oral single doses 1–800 mg; or 30, 100, 300, 500 or 800 mg daily for 14 days	Maximum placebo-adjusted reductions of 63–65% at ≥100 mg/day (days 14–15; apo(a)-based assay).	No SAEs; mostly mild events. Transient decrease in plasminogen activity, maximal about 14% in the 500 mg group, returning to baseline after treatment.
Nicholls et al. 2025 [152] KRAKEN Phase 2 NCT05563246	Muvalaplin	*n* = 233; age ≥ 40 years; Lp(a) ≥ 175 nmol/L; ASCVD, type 2 diabetes or FH	Oral 10, 60 or 240 mg daily for 12 weeks	Week 12 placebo-adjusted reductions: 47.6%, 81.7%, 85.8% by intact-Lp(a) assay; 40.4%, 70.0%, 68.9% by apo(a)-based assay, respectively.	Overall AE incidence similar. Discontinuations more frequent at 240 mg; liver enzyme elevations included one >10× ULN with confounding conditions/medication. No significant plasminogen-activity effect.

Notes: All listed interventional studies used randomized placebo-controlled designs. Doses and schedules are trial regimens. Placebo-adjusted changes can exceed 100% in magnitude and do not imply a negative measured concentration. Abbreviations: AE, adverse event; apo(a), apolipoprotein(a); ASCVD, atherosclerotic cardiovascular disease; CRP, C-reactive protein; CV, cardiovascular; CVD, cardiovascular disease; FH, familial hypercholesterolemia; Lp(a), lipoprotein(a); q, every; SAE, serious adverse event; SC, subcutaneous; ULN, upper limit of normal; wk, week.

## Data Availability

No new data were created or analyzed in this study. Data sharing is not applicable to this article.

## Abbreviations

The following abbreviations are used in this manuscript:

ABCA1	ATP-binding cassette transporter A1
ACC	American College of Cardiology
AHA	American Heart Association
ACS	acute coronary syndrome
ACTA2	actin alpha 2, smooth muscle
AE	adverse event
AI	artificial intelligence
AP-1	activator protein 1
apo(a)	apolipoprotein(a)
apoA-I	apolipoprotein A-I
apoB	apolipoprotein B
apoB-100	apolipoprotein B-100
AS	atherosclerosis
ASCVD	atherosclerotic cardiovascular disease
ASO	antisense oligonucleotide
CAC	coronary artery calcium
CAD	coronary artery disease
CCA	common carotid artery
CCL28	C-C motif chemokine ligand 28
CCR7	C-C chemokine receptor type 7
CCS	chronic coronary syndrome
CCTA	coronary computed tomography angiography
CC	cholesterol crystal
CD	cluster of differentiation
CEC	cholesterol efflux capacity
CI	confidence interval
CMCS	Chinese Multi-Provincial Cohort Study
CNN1	calponin 1
CRP	C-reactive protein
CV	cardiovascular
CVD	cardiovascular disease
CXCL10	C-X-C motif chemokine ligand 10
DG	diacylglycerol
ECM	extracellular matrix
EGR1	early growth response 1
ELK1	ETS transcription factor ELK1
EPC	endothelial progenitor cell
ER	endoplasmic reticulum
ERK	extracellular signal-regulated kinase
ESC	European Society of Cardiology
EAS	European Atherosclerosis Society
FADD	Fas-associated death domain protein
FAK	focal adhesion kinase
Fas	Fas cell surface death receptor
FH	familial hypercholesterolemia
GalNAc	N-acetylgalactosamine
HDL	high-density lipoprotein
hs-CRP	high-sensitivity C-reactive protein
ICA	internal carotid artery
IL	interleukin
IPH	intraplaque hemorrhage
IPN	intraplaque neovascularization
IVUS	intravascular ultrasound
JNK	c-Jun N-terminal kinase
KIV	kringle IV
KIV-2	kringle IV type 2
KLF4	Kruppel-like factor 4
KV	kringle V
LCBI	lipid core burden index
LDL	low-density lipoprotein
LDL-C	low-density lipoprotein cholesterol
Lp(a)	lipoprotein(a)
LPA	lipoprotein(a) gene
Lp-PLA2	lipoprotein-associated phospholipase A2
LPC	lysophosphatidylcholine
LRNC	lipid-rich necrotic core
Ly6C	lymphocyte antigen 6 complex locus C
Mac-1	leukocyte integrin αMβ2
M1	classically activated macrophage
MACE	major adverse cardiovascular events
MAPK	mitogen-activated protein kinase
maxLCBI4mm	maximum LCBI over a 4 mm segment
MD	mitochondrial dysfunction
MDCTA	multidetector computed tomography angiography
MEK	mitogen-activated protein kinase/ERK kinase
MeSH	Medical Subject Headings
MI	myocardial infarction
MIAT	myocardial infarction associated transcript
MMP	matrix metalloproteinase
MMP-9	matrix metalloproteinase-9
MRI	magnetic resonance imaging
mRNA	messenger RNA
MYH11	myosin heavy chain 11
NF-κB	nuclear factor kappa B
NIRS	near-infrared spectroscopy
NIRS-IVUS	near-infrared spectroscopy-intravascular ultrasound
NLR	NOD-like receptor
NLRP3	NLR family pyrin domain containing 3
NOX2	NADPH oxidase 2
OCT	optical coherence tomography
OR	odds ratio
OxPL	oxidized phospholipids
p38	p38 mitogen-activated protein kinase
p53	tumor protein p53
PAV	percent atheroma volume
PB	plaque burden
PCAT	pericoronary adipose tissue
PCI	percutaneous coronary intervention
PCSK9	proprotein convertase subtilisin/kexin type 9
PLG	plasminogen
PoLA	Polish Lipid Association
RAF	rapidly accelerated fibrosarcoma kinase
ROS	reactive oxygen species
RR	relative risk
SAE	serious adverse event
SC	subcutaneous
siRNA	small interfering RNA
SMC	smooth muscle cell
TAGLN	transgelin
TCFA	thin-cap fibroatheroma
TF	tissue factor
TGF-β	transforming growth factor-β
TIA	transient ischemic attack
TLR	Toll-like receptor
TNF	tumor necrosis factor
TREM2	triggering receptor expressed on myeloid cells 2
ULN	upper limit of normal
VLDL	very-low-density lipoprotein
VLDL-R	very-low-density lipoprotein receptor
VSMC	vascular smooth muscle cell
wk	week
